# Lack of a type-2 glycosyltransferase in the fish pathogen *Flavobacterium psychrophilum* determines pleiotropic changes and loss of virulence

**DOI:** 10.1186/s13567-014-0124-5

**Published:** 2015-01-13

**Authors:** David Pérez-Pascual, Esther Gómez, José A Guijarro

**Affiliations:** Área de Microbiología, Departamento de Biología Funcional, Facultad de Medicina, IUBA, Universidad de Oviedo, 33006 Oviedo, Spain; Present address: INRA, Institut Micalis, Équipe Peptides et Communication Bactérienne, Domaine de Vilvert, bâtiment 526, 78352 Jouy-en-Josas cedex, France

## Abstract

*Flavobacterium psychrophilum* is an important fish pathogen, responsible for Cold Water Disease, with a significant economic impact on salmonid farms worldwide. In spite of this, little is known about the bacterial physiology and pathogenesis mechanisms, maybe because it is difficult to manipulate, being considered a fastidious microorganism. Mutants obtained using a Tn*4351* transposon were screened in order to identify those with alteration in colony morphology, colony spreading and extracellular proteolytic activity, amongst other phenotypes. A *F. psychrophilum* mutant lacking gliding motility showed interruption of the FP1638 locus that encodes a putative type-2 glycosyltransferase (from here on referred to as *fpgA* gene, *Flavobacterium psychrophilum* glycosyltransferase). Additionally, the mutant also showed a decrease in the extracellular proteolytic activity as a consequence of down regulation in the *fpgA* mutant background of the *fpp2-fpp1* operon promoter, responsible for the major extracellular proteolytic activity of the bacterium. The protein glycosylation profile of the parental strain showed the presence of a 22 kDa glycosylated protein which is lost in the mutant. Complementation with the *fpgA* gene led to the recovery of the wild-type phenotype. LD_50_ experiments in the rainbow trout infection model show that the mutant was highly attenuated. The pleiotropic phenotype of the mutant demonstrated the importance of this glycosyltranferase in the physiology and virulence of the bacterium. Moreover, the fpgA mutant strain could be considered a good candidate for the design of an attenuated vaccine.

## Introduction

*Flavobacterium psychrophilum* is a Gram-negative bacterium and it is a member of the *Cytophaga-Flavabacterium-Bacteroides* (CFB) group. This microorganism is the etiological agent of Cold Water Disease (CWD) (as well as rainbow trout fry syndrome), one of the most important infectious pathologies, causing significant economic losses in salmonids on fish farms worldwide. Outbreaks occur at temperatures below 14 °C and mainly affect fingerlings. Currently there are no efficient commercial vaccines to protect against CWD. Besides, other approaches such as the use of immunostimulants, probiotics or lytic bacteriophages do not provide sufficient infection control. Therefore, disease management is based on fish monitoring strategies and the use of antimicrobial therapy [1,2].

In spite of being considered a fastidious bacterium because it is difficult to isolate and manipulate [3], several advances have been reported during the last few years in culture [4], experimental infection techniques [5], typing [6-9], molecular diagnosis [10-15] and genomics [16]. Furthermore, genetic manipulation systems based on transposition mutagenesis [17] and disruption of the specific genes by homologous recombination have been developed [18]. However, the mechanisms of pathogenicity of this bacterium are still unknown and little data exist about the genetic basis of virulence [19,20].

Even though protein glycosylation was once considered to be a specifically eukaryotic phenomenon, nowadays it is clear that it is widespread in prokaryotes [21,22]. In fact, several glycoproteins described in Gram-negative pathogenic bacteria have been associated with virulence [23,24]. In the case of *F. psychrophilum*, a glycosylated protein, OmpA, has been purified and characterized [25,26]. In addition, 35 genes encoding putative glycosyltransferases have been identified in the genome of strain JIP02/86 [16]. Moreover, some members of the CFB group showed glycosylated proteins [27,28]. In particular, *Bacteroides fragilis,* forming a relevant part of the human intestinal microbiota*,* presented, at least eight glycoproteins which are related to the competitive colonization of mammalian intestines [28,29]. All of this suggests a relevant role of glycosyltransferases in the biology of bacteria belonging to this group.

The ability of pathogenic bacteria to cause disease depends to a large extent on the expression of specific gene-encoding factors which help them to invade the host tissues, develop the infection and evade host defenses. Amongst other characteristics, bacterial motility [30-32] and extracellular proteolytic activity [33] are considered to be involved in the infection caused by different Gram-negative bacteria. With the aim of achieving a deeper insight into the pathogenesis mechanisms of *F. psychrophilum*, a set of mutants deficient in gliding motility and extracellular proteolytic activity were isolated using the Tn*4351*-mutagenesis system [19]. In this study, a mutant with a change in the FP1638 locus was further analyzed. The interrupted gene, which encodes a type-2 glycosyltransferase, was found to be involved in the glycosylation of a 22 kDa protein. The modification of protein glycosylation in the mutant strain caused, amongst other effects, high attenuation of the virulence of the bacterium.

## Material and methods

### Bacterial strains and growth conditions

The strains, plasmid and primers used in this study are listed in Table 1. *Escherichia coli* strains S17-1 *λpir* [34] and BW19851 [35] were used to transfer DNA into *F. psychrophilum* THC02/90. *E. coli* strains were grown at 37 °C in 2 × TY medium (10 g of tryptone per liter, 10 g of yeast extract per liter, 5 g of NaCl per liter) with 20 g of agar per liter added for solid medium. *F. psychrophilum* THC02/90 was grown at 12 °C or 18 °C in nutrient broth (NB; Pronadisa, Madrid, Spain) or NBF [36]. Nutrient agar (NA: NB containing 1.5 g of agar/L), or nutrient agar charcoal (NAC; NA supplemented with 0.05% activated charcoal), were used for solid cultures as previously described [36]. Growth in liquid culture was carried out at 250 rpm and 12 °C and determined by measuring OD_525nm_ at different times. Stock cultures were kept in NB containing 25% glycerol at −80 °C. To observe colony spreading, *F. psychrophilum* strains were grown as previously described in 1/6NA [37]. Extracellular proteolytic activity on solid medium was visualized using NBF containing 1.5 g/mL of agar and supplemented with 0.75% gelatin [19]. Azocasein assays were performed according to Secades et al., [38] where one unit of enzyme activity [32] was defined as the amount that yielded an increase in the OD_420nm_ of 0.01 in two hours at 30 °C. For selective growth of *E. coli* S17-1 *λpir*, 50 μg/mL streptomycin was used and transformants were selected with 100 μg/mL ampicillin. Selection of *F. psychrophilum* transconjugants was carried out with 10 μg/mL erythromycin and 10 μg/mL tetracycline.

**Table 1:** **Bacterial strains, plasmids and primers used in this study**

**Plasmid, bacterial strain or primer**	**Description or sequence**	**Source or reference**
Plasmids^a^		
pEP4351	*Ori* R6K dependent protein pir,; RP4 oriT; Cm^r^ Tc^r^ (Em^r^); Tn*4351* vector transfer.	[39]
pCP23	ColE1 ori, (pCP1 ori), Ap^r^ (Tc^r^), *E. coli*–*F. psychrophilum* shuttle plasmid	[40]
pCP23-fpgA	*FP1638* gene, derived from pCP23.	This study
pCP23-Gfpp2	pCP23-G carrying *fpp2-fpp1* promoter	[41]
Bacterial strains		
*F. psychrophilum*		
THC02/90	Wild-type.	[42]
fpgA^−^	*FP1638* mutant created by Tn*4351* transposition.	This study
fpgA+	Mutant ΔFP1638 carrying pCP23-FP1638 plasmid, complemented strain.	This study
THC02/90-G	THC02/90 strain carrying pCP23-G plasmid	[41]
THC02/90 -fpp2	THC02/90 strain carrying pCP23-Gfpp2 plasmid	[41]
fpgA^−^ -fpp2	ΔFP1638 strain carrying pCP23-Gfpp2 plasmid	[41]
*E. coli*		
S17-1 *λpir*	λ*pir hsdR pro thi*; RP4-2 Tc::Mu Km::Tn*7*	[34]
BW19851	RP4-2*tet*::Mu-1*kan*::Tn*7* integrant; _*uidA*::*pir*_ *recA1 hsdR17 creB510 endA1 zbf-5 thi*	[35]
Primers^b^		
26-F	5′ACTG**GGATCC**AGTTTTAAGCCCGCAAA 3′	This study
26-R	5′ ACTG**CTGCAG**CAATGAACTTCGTCTTG 3′	This study
TN-1	5′ GGACCTACCTCATAGACAA 3′	[19]
IS4351-F	5′ TCAGAGTGAGAGAAAGGG 3′	[19]
promfpp2-F	5′ ATCA**GGATCC**GAGCACTACACTTTCTAGA 3′	[41]
promfpp2-R	5′ GATT**GGATCC**TGTTCGGTAGTGTAGCA 3′	[41]

^a^Antibiotic resistance phenotypes: ampicillin, Ap^r^; tetracycline, Tc^r^; erythromycin, Emr^b^. Antibiotic resistance phenotypes and other features listed in parentheses are those expressed by *F. psychrophilum* but not by *E. coli*.

^b^Restriction sites for cloning are in bold.

### DNA technology

Genomic DNA extractions were performed with the Gen Elute Bacterial DNA (Sigma-Aldrich Co., St-Louis, MO, USA) extraction kit. Plasmid DNA was purified with the Gen Elute Plasmid miniprep (Sigma-Aldrich Co.) kit.

PCR amplification products were separated on 1.5% agarose gels and bands were purified with the Illustra™ GFX, PCR DNA and the Gel Band Purification Kit gel extraction system.

### Identification of Tn*4351* interrupted locus and sequencing of the surrounding DNA region

The insertion of Tn*4351* into the genome of the *fpgA* mutant strain was confirmed by Southern blot analysis. Total DNA from the mutant and parental strains was isolated and digested with *Hind*III and *Xba*I restriction enzymes. After agarose gel electrophoresis, DNA fragments were transferred to a nylon membrane (Amershan Bioscience, Uppsala, Sweden) and fixed with UV irradiation. A DIG DNA labeling and detection kit (Roche, Basel, Switzerland) was used to prepare the probe and to perform hybridization. As a probe, a 6.2 kb *Sal*I fragment from pEP4351 containing Tn*4351* transposon was used [19].

To isolate and sequence the genomic DNA flanking the Tn*4351* chromosomal insertion, DNA of the mutant strain was digested with *Hind*III followed by a re-ligation process. The resulting circular molecules were used as a template to amplify by inverse PCR the sequences adjacent to the Tn*4351* insertion site using a specific pair of primers TN-1/IS4351-F (Table 1) and the Certamp long amplification kit (Biotools B&M Laboratories, Madrid, Spain).

Automated fluorescence sequencing of the PCR amplified products was performed at the Oviedo University DNA analysis facility using BigDye 3.1 Terminator chemistry on an ABI PRISM 3100 Genetic Analyzer platform (Applied Biosystems, CA, USA). Sequences were compared to databases using the Basic Local Alignment Search Tool (BLAST) from the National Center for Biotechnology Information (NCBI).

### Complementation of the *fpgA* gene mutation

To complement the fpgA mutant, the DNA sequence corresponding to the encoding gene was amplified from the parental strain by PCR, using 26-F and 26-R primers and the Expand Long Template PCR System (Roche), obtaining a fragment of 1105 pb (Table 1). *Bam*HI and *Pst*I restriction sites were introduced into the sequences of 26-F and 26-R respectively, in order to clone the PCR product into the plasmid pCP23 that has promoter activity [41] (Table 1). The resulting plasmid was designated pCP23-fpgA (Table 1). Transfer of pCP23-fpgA to *F. psychrophilum* was carried out by conjugation as previously described [17] and pCP23-fpgA was recovered from the transconjugants, digested with *Bam*HI and *Pst*I and analyzed by agarose gel electrophoresis to confirm the presence of the insert.

### Transcriptional fusion analysis based on *gfp* reporter gene

The previously constructed pCP23-Gfpp2 (Table 1), derived from the GFP-based reporter vector pCP23-G [41], was used for the analysis of the transcriptional activity of the *fpp2-fpp1* operon [18] in the context of wild-type and fpgA^−^ strains. The pCP23-Gfpp2 plasmid was introduced by transformation in *E. coli* S17 *λpir* and further conjugated into *F. psychrophilum* fpgA^−^ and wild-type strains as described by Álvarez et al. [17], originating the fpgA^−^-fpp2 and THC02/90-fpp2 strains (Table 1). For flow cytometry analysis, 5 mL of NB supplemented with tetracycline were inoculated with 50 μL of mid-exponential-phase cultures from fpgA^−^-fpp2 and THC02/90-fpp2 strains. The THC02/90-G strain (Table 1) was used as the fluorescence emission negative control. They were incubated at 12 °C in NB supplemented with 10 mM CaCl_2_ in an orbital shaker at 250 rpm. Then, early-stationary phase cultures were washed once with PBS and resuspended in 500 μL of PBS prior to fluorescence emission analysis. Promoter expression quantification was assessed in a flow cytometer (Beckman Coulter Cytomics FC 500) with 488 Ar and 633 HeNe lasers. Green fluorescence was detected on FL-14 channel (505–545 nm) and 10 000 events were acquired from each sample. All cultures were analyzed in triplicate. Fluorescence emission, with and without calcium in culture media, was assessed in a Student’s *t*-test. Differences were considered significant when *p*-value < 0.05.

### LD_50_ determination

For LD_50_ experiments rainbow trout (*Oncorhynchus mykiss*) weighing between 5 and 7 g were kept in 60-liter tanks at 14 ± 1 °C in continually flowing dechlorinated water.

On arrival at the aquarium, the fish were acclimatized to experimental conditions and 5% were selected randomly and screened for any bacteria. Samples from liver, muscle, blood and spleen were taken and plated onto permissive media (TSA and NAC) and incubated at 18 °C for 72 h. If bacterial growth was observed in these samples, the whole batch of fish was discarded for further experiments. Cultures of *F. psychrophilum* parental and fpgA^−^ strains were grown to exponential phase, harvested by centrifugation and washed twice with PBS. Cells were resuspended in PBS and serial dilutions were prepared. Groups of 10 fish were challenged by intramuscular injection of 50 μL of dilutions containing 10^3^-10^9^ colony forming units (CFU) for the parental strain and 10^6^-10^9^ CFU for the fpgA^−^ mutant strain, and LD_50_ was calculated ten days post-infection according to the method of Reed and Muench [43]. In the control group 10 fish were injected with 50 μL of PBS. To verify the presence of *F. psychrophilum* in dead fish, samples obtained from muscle (the injection zone), brain, liver and eyes were plated onto NAC plates (supplemented with the corresponding antibiotic for mutant strain) and incubated for 72 h at 18 °C. The assay was qualitative and was performed in order to confirm that the dead fish had a massive presence of the bacterium in different organs due to the development of septicemia. After culture, PCR was routinely used for the identification of the bacterium, using specific primers [13,19]. The results are the average of two independent experiments. Animal experiments were performed in accordance with the European legislation governing animal welfare, and they were authorized and supervised by the Animal Experimentation Ethics Committee of Universidad de Oviedo.

### Analysis of protein glycosylation

The wild-type, fpgA^−^ and fpgA^−^ carrying pCP23-fpgA plasmid (from here, fpgA^+^) strains were grown in NB for 120 h at 12 °C, harvested by centrifugation (10 000 rpm, 10 min) and washed twice with Tris–HCl (50 mM pH 6.8). Pelleted cells were re-suspended in 1 mL of the same buffer and cells were broken by ten 12-s sonication with 1 min intervals in an ice bath. Then, the suspension was centrifuged (12 000 rpm, 20 min, 4 °C) and aliquots of the supernatant were used for protein analysis by 14% SDS-PAGE according to the Laemmli methods. Proteins were visualized by silver and Coomassie brilliant blue staining. Glycosylated proteins were detected using the Pro-Q Emerald 300 glycoprotein staining kit (Life Technologies, Carlsbad, CA, USA), according to the manufacturer’s instructions. The effect of proteinase K (50 μg/mL) and trypsin (12 μg/mL) on the glycosylated proteins was assessed by incubation of the total protein extract from the wild-type strain at 60 °C for 1 h before separation by SDS-PAGE. When a mixture of both enzymes was used, the reaction with proteinase K was carried out first. Then, an additional incubation in the presence of trypsin was performed under the same conditions. Controls without the presence of proteases were carried out simultaneously in all cases.

### Genetic analysis

The BLAST program was used to compare protein sequences and Simple Modular Architecture Research Tools (SMART) [44] for detecting conserved domains. MotifScan software from MyHits was used to identify the motifs present in each sequence. The carbohydrate-active enzymes (CAZy) [45] bank was used to classify the glycosyltransferase. The ProtParam program [46] was used for molecular mass computation and SignalP3.0 [47] to predict the possible location of a signal peptide cleavage site.

## Results

### Analysis of the Tn*4351*-disrupted sequence of *fpgA*

A set of *F. psychrophilum* mutants were obtained by Tn*4351* insertional mutagenesis as described by Álvarez et al. [17] and then in order to study the gliding-virulence relationship, a mutant strain, defective in colony spreading, was further analyzed. Southern blot analysis of this mutant revealed a single transposon insertion in its genome (data not shown). The DNA sequence surrounding the transposon insertion was obtained by inverse PCR as described by Álvarez et al. [19]. The disrupted sequence for the isogenic mutant corresponds to the FP1638 locus (Gen Bank accession no. NC_009613) of the *F. psychrophilum* genome [16], a gene of 965 bp that encodes for a 319-amino-acid protein with a predicted mass of 38 081 Da. The transposon was inserted after nucleotide 774. The protein function corresponds to a type-2 glycosyltransferase since it presents a catalytic domain between amino acids 4 and 178, characteristic of the subfamily 2 glycosyltransferases [16]. It also shows a high level of similarity with this kind of enzyme from a variety of species such as *Dyadobacter fermentans* DSM 18053 (Gen Bank accession no. YP_003088235.1), *Methanosarcina barkeri* Fusaro strain WcaA (Gen Bank accession no. YP_303801.1) and *Pelosinus fermentas* (Gen Bank accession no. WP_0079027).

**Figure 1:**
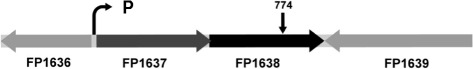
**Genomic organization of the region surrounding the FP1638 (** ***fpgA*** **) gene in** ***F. psychrophilum.*** The direction of transcription is indicated by arrows. The position of a putative promoter (P) involved in the regulation of both the FP1637 and FP1638 gene (separated by 4 bp) is indicated. Transposon insertion is located at position 774 of the *fpgA* locus.

The gene appears in the same orientation as FP1637 (Gen Bank accession no. NC_009613.3) and is separated from it by 4 bp in the genome of strain JIP02/86 of *F. psychrophilum* [16]. An identical situation was defined by PCR and further sequencing for the *F. psychrophilum* THCO2/90 strain. FP1637 codifies a protein of unknown function which exhibits high sequence identity to predicted WbhW protein of *E. coli,* similar to a glycosyltransferase enzyme [16]. A sigma 70-like promoter sequence was located twenty base pairs upstream of the FP1637 start codon (Figure 1). These results indicate that FP1637 and FP1638 loci could form an operon regulated by the promoter region mentioned.

### Phenotypic characterization of fpgA^−^ strain and complementation assays

**Figure 2:**
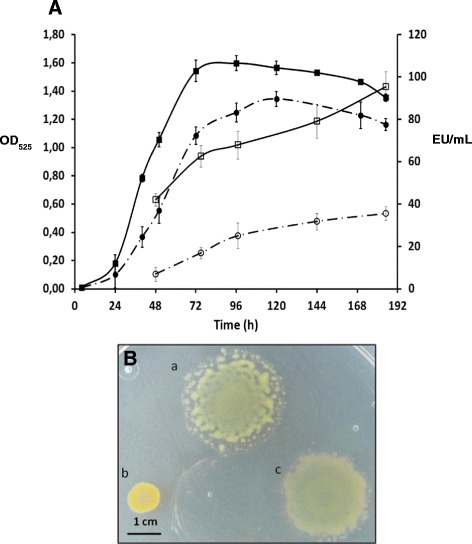
**Growth, extracellular proteolytic activity and colony spreading of** ***F. psychrophilum*** **wild-type and fpgA** ^**−**^ **strains.** **(A)** Growth curve at 12 °C in NBF medium, monitored by determining OD_525_. (■) wild-type and (●) fpgA^−^ strains. Extracellular caseinolytic activity in cell-free supernatants was determined with azocasein as described by Secades et al. [38]. (□) wild-type and (○) fpgA^−^ strains. EU, enzyme units. **(B)** Colony spreading of *F. psychrophilum* strains grown on 1/6NA for 96 h at 20 °C according to Pérez-Pascual et al. [37]. **(a)** wild-type; **(b)** fpgA^−^; and **(c)** fpgA^+^ (complemented strain).

With the aim of making a deeper analysis of the fpgA^−^ strain, different phenotypic characteristics were studied. At 12 °C, the mutant presented a slightly lower growth rate and a decreased final cell density in comparison with the parental strain (Figure 2A). When extracellular proteolytic activity was analyzed, a significant decrease was observed in relation to the parental strain (Figure 2A).

In order to go further into the cause of the decrease in the extracellular proteolytic activity of the fpgA^−^ strain, the pCP23-Gfpp2 plasmid, containing a transcriptional fusion between the *fpp2* promoter, regulating the main gene driving caseinolytic activity in the bacterium under in vitro conditions [18], and the *gfpmut3* gene, was introduced into fpgA^−^. Strain THC02-90/G with the plasmid pCP23-G harboring a promoterless *gfpmut3* gene was utilized as a negative control and no fluorescence emission could be detected under the growth conditions tested. THC02/90-*fpp2* and *fpgA*^*−*^*-fpp2* showed 117.02 ± 49.42 fluorescence relative units (fru) and 17.46 ± 3.00 fru, respectively (*p*-value 0.013). Therefore, promoter expression quantification by flow cytometry shows that, in the fpgA^−^ background, *fpp2* promoter expression was around 85% lower than that found in the parental strain in the final-exponential phase of the growth curve.

Additionally, fpgA^−^ showed colony spreading defects, forming non-spreading colonies with rounded edges, when grown on 1/6NA medium, whereas the parental strain presented the characteristic high spreading phenotype (Figure 2B). Complementation experiments using pCP23-derived plasmid show that the presence of the plasmid pCP23-*fpgA* in fpgA^−^ restored both colony spreading (Figure 2B) and extracellular proteolytic activity (89.2 EU/mL at 168 h of incubation).

### Protein glycosylation profile of wild-type, mutant and complemented strains

**Figure 3:**
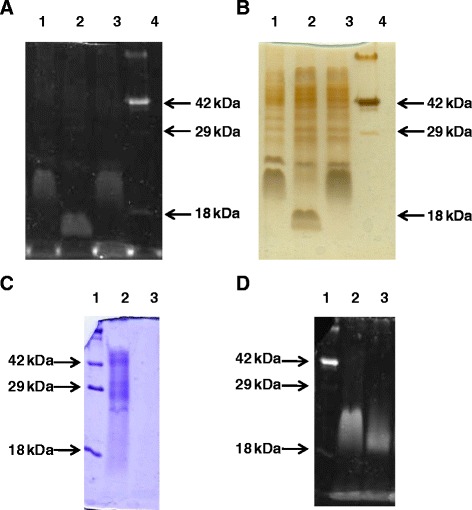
**SDS-PAGE and gel staining of glycosylated proteins extracted from** ***F. psychrophilum*** **wild-type, fpgA** ^**−**^ **, and fpgA** ^**+**^ **(complemented fpgA** ^**−**^ **) strains.** Total extracted proteins were separated by 14% SDS-PAGE gels that were **(A, D)** Pro-Q Emerald specific protein glycosylation; **(B)** silver; and **(C)** Coomassie blue, stained. **(A)** and **(B)**: lane 1, parental; lane 2, fpgA-; and lane 3, fpgA+. In the right of each image, the position of Candy Cane (Life Technologies, Carlasbad, Cal.) with the 42 kDa and 18 kDa glycosylated proteins is indicated (lane 4). **(C)** and **(D)**: lane 2, wild-type strain; lane 3, wild-type strain extract treated with proteinase K (similar results were obtained when trypsin and a mixture of both enzymes were used). Lane 1, Candy Cane (Life Technologies, Carlasbad, Cal.) molecular mass markers.

Taking account of the predicted FpgA protein function, an analysis of protein glycosylation profiles of both parental and mutant strains was carried out in order to further characterize its role. The parental strain presented a wide and diffuse glycosylated band of around 22 kDa, whereas fpgA^−^ lacked this band, showing a new one of around 18 kDa (Figure 3A). This band could also be observed after silver staining (Figure 3B). The treatment of the extracts with different proteases show that in all proteolytic processes a new glycosylated band of about 18 kDa appeared (Figure 3C and D), suggesting a proteinaceous nature for the 22 kDa product. Different attempts were carried out in order to purify and further identify the peptide amino acid sequence of the 22 kDa glycosylated protein by mass spectrometry analysis but, unfortunately, they were unsuccessful. The complementation of the fpgA^−^ strain with the pCP23 plasmid harboring the *fpgA* gene resulted in the recovery of the glycosylation profile of the wild-type strain, with the presence of a band of 22 kDa (Figure 3A).

### LD_50_ experiments

In order to evaluate the effect of the *fpgA* mutation in the virulence of *F. psychrophilum*, LD_50_ experiments were carried out on rainbow trout. Ten days post-infection, the LD_50_ value was 2.63 × 10^6^ CFU for the wild-type strain. Under the same conditions, fpgA^−^ showed LD_50_ values higher than 10^9^ CFU. Thus, the mutant strain for the *fpgA* locus could be considered as being highly attenuated compared to the wild-type strain. Additionally, visual examination for characteristic erosive skin lesions around the injection site showed that fpgA^−^ infected fishes did not present any kind of tissue damage comparable to that seen in parental strain infections (data not shown). Cultures on NAC medium of samples from the different organs of dead fish show the presence exclusively of the characteristic yellow-pigmented colonies of *F. psychrophylum*, as was expected in the case of septicemia. Routine PCR analyses show that these colonies corresponded to *F. psychrophilum* (data not shown).

## Discussion

*F. psychrophilum* mutants obtained by Tn*4351* transposition were selected on the basis of the presence of alteration in colony spreading [19]. The fpgA^−^ strain presented the disruption in a gene encoding a protein with a glycosyltransferase function. Its genetic context strongly suggests that the *fpgA* gene forms an operon with the FP1637 gene, due to the short distance between them (4 bp) and the presence of a promoter sequence upstream of FP1637.

Extracellular proteolytic activity and colony spreading were affected in fpgA^−^. Complementation experiments show that the *fpgA* gene is responsible for both effects. Involvement of glycosyltransferases in bacterial motility is not limited to *F. psychrophilum*; these kind of proteins usually positioned in the outer membrane, generate polysaccharides implicated, for example, in bacterial attachment [48], an essential step for cell movement by gliding. In addition, these enzymes participate in the slime biosynthesis processes in *Myxococcus xanthus* and, consequently, are involved in the adventurous motility (A motility), where some authors proposed that the bacterium glides over a trail of polysaccharide [49,50]. In the same way, the disruption of three different glycosyltransferases in the marine bacterium *Synechococcus sp.* resulted in a non-swimming phenotype in mutant strains, indicating that these enzymes are required for motility [51].

Under the assayed conditions, most of the extracellular proteolytic activity detected from the wild-type strain is caused by the metalloproteases Fpp1 and Fpp2 whose encoding genes *fpp2-fpp1* form an operon [18]. As shown by GFP-based regulation experiments, the expression of this operon was found to be lower in the fpgA^−^ context than that of the wild-type strain. This result suggests that the decrease of extracellular proteolytic activity detected in the *fpgA* mutant could be a consequence of down-regulation of, at least, the *fpp2-fpp1* operon. As occurring in *F. johnsoniae,* mutation in genes involved in gliding (*gld* genes) determined a change in the secretion of extracellular enzymes [52-54]. Until now, it has not been possible to establish a relationship between these two phenomena. However, it seems that a change in regulation affecting extracellular proteolytic activity occurs in parallel to the loss of the ability to move, rather than a physical interaction between the gliding machinery and the secretion systems.

The disruption of the *fpgA* gene, encoding a glycosyltransferase, caused an altered glycosylation profile in the mutant strain. While the wild-type strain presented a glycosylated element of 22 kDa, fpgA^−^ shows an 18 kDa glycoconjugate. In any case, care must be taken when assigning a molecular mass size by SDS-PAGE in glycosylated proteins. The effect of proteolytic enzymes on the 22 kDa glycosylated band strongly suggests that it has a proteinaceous nature. The fact that a glycosylated core of 18 kDa is still present after the proteolytic treatments could be a consequence of steric impediments that might make it difficult for the proteases to gain access to the whole protein; currently, it is suggested that the addition of carbohydrates to a protein could be related to major resistance to the proteolysis process [23,55]. Taking into account that the function ascribed to FpgA corresponds to a glycosyltransferase, the results suggest that the 22 kDa protein has, at least, double glycosylation modification. One of them is probably carried out by FpgA, since the interruption of the coding locus does not involve lack of glycosylation, but a reduction of the molecular mass of the 22 kDa glycosylated protein to 18 kDa. In this sense, *B. fragilis* a member of the CFB group, presents a protein with a triple glycosylation modification [29]. Complementation experiments confirm this hypothesis since, the introduction of the *fpgA* gene into fpgA^−^ led to the recovery of the 22 kDa glycosylated protein. It is interesting to point out that several members of the CFB group show differential glycoprotein production during their growth [28]. Thus, in *B. fragilis* 8 glycoproteins have been identified [29]; in *Elizabethkingia meningoseptica* (former *Flavobacterium meningosepticum*) [56,57] and *F. columnare*, several secreted proteins showed glycosylations [58]. These data, together with the glycosylation of the *F. psychrophilum* outer membrane protein OmpA [25,26] and the proportion of putative glycosyltransferases present in the genome of *F. psychrophilum,* strongly suggest a greater relevance of this type of enzyme in the physiology of the CFB group in relation to other bacteria, as already proposed by Fernandez-Gomez et al. [48]. The importance of the glycosylation process in the virulence of the bacterium is clear since loss of virulence in the mutant strain was definitive. The pleiotropic phenotype of the mutant strain makes the determination of the phenotypic characteristic responsible for the loss of pathogenecity, difficult. Intriguingly, in many Gram-negative pathogenic bacteria most glycoproteins were associated with pathogenesis [59]. This is the case for the glycosylation of the HWM1 adhesin of *Haemophilus influenzae* [60], the flagella in *Campylobacter jejuni* [59,61,62] and the protein-forming type IV pili in *Neisseria meningitidis* [63], amongst others. The lack of virulence seen in the fpgA^*−*^ strain indicates that this strain could be a suitable candidate for the development of attenuated vaccines for the prevention of CWD. According to the pleiotropic effect generated by the mutation of the *fpgA* gene, it could not be ruled out that FpgA could be involved in a chaperon system as suggested for some glycosylated proteins in *B. fragilis* [29].

This work, together with recent data, indicates that protein glycosylation in prokaryotes and, in particular, in the CFB group could play an important role on the biology of these microorganisms, as previously suggested [28,48]. All of this could suggest that this glycosyltransferase has a regulatory role. Additionally, the avirulent mutant strain could be considered a good candidate to be used as a live vaccine in order to control the CWD.
